# Beneficial Effects of Probiotic Treatment on Gut Microbiota in Very Low Birth Weight Infants

**DOI:** 10.1155/2019/3682836

**Published:** 2019-10-17

**Authors:** Yue-feng Li, Chuan-rui Zhu, Xue-lei Gong, Hui-ling Li, Li-kuan Xiong, Ke-jian Wang, Guo-Sheng Liu

**Affiliations:** ^1^Department of Pediatrics, The First Affiliated Hospital, Jinan University, Guangzhou 510630, China; ^2^Department of Pediatrics, Shenzhen Luohu Maternity and Child Health Hospital, 518019, China; ^3^Department of Neonatology, Bao'an Maternal and Child Health Hospital, Shenzhen 518133, China; ^4^Central Laboratory, Bao'an Maternal and Child Health Hospital, Shenzhen 518133, China; ^5^Lin He's Academician Workstation of New Medicine and Clinical Translation, The Third Affiliated Hospital, Guangzhou Medical University, Guangzhou 510150, China

## Abstract

The very low birth weight (VLBW) infant is at great risk for marked dysbiosis of the gut microbiota. In the present study, a total of 36 VLBW infants were randomly divided into two groups, who were treated with combined probiotics and placebo, and 72 fecal specimens on days 14 and 28 of life were collected from them. Finally, 32 fecal specimens extracted from 16 preterm VLBW infants were qualified and analyzed using 16S rRNA gene sequencing. The primary outcome was to evaluate the change of gut microbiota in VLBW infants after combined probiotic supplement. The secondary outcome was to analyze the correlation gut microbial composition and levels of cytokines. We found that probiotic treatment, but not placebo, decreased the *α*-diversity of gut microbiota in VLBW infants. At the phylum level, probiotic treatment strongly increased the abundance of *Firmicutes*, whereas that of *Proteobacteria* was significantly reduced. At the family level, *Streptococcaceae* and *Lactobacillaceae* became prevalent after probiotic treatment, while the relative abundance of *Enterobacteriaceae* was reduced in the meantime. Most notably, significant correlations were observed between *Lactobacillaceae* abundance and serum cytokine levels. Further studies are required to shed more light on the characteristics of gut microbiota of VLBW neonates. And the modulation of microbiota should be considered to improve the survival rate of VLBW infants.

## 1. Introduction

Due to recent advances in the neonatal intensive care, the survival rates of extremely preterm infants were significantly increased over the last 20 years [1]. However, high death and morbidity were still observed in infants born >26 weeks in a multicenter survey in China [2]. Thus, improvements in survival have not been accompanied by proportional reductions in the incidence of disability in this population. Necrotizing enterocolitis (NEC) is a common gastrointestinal emergency and leading cause of morbidity and mortality in extremely preterm infants. Many meta-analyses of RCTs had shown that oral probiotics effectively reduce NEC and death [3, 4]. In addition, an update meta-analyses study showed that multiple strain probiotics could be more effective in preventing NEC and death in extremely preterm infants [5], but it is still unclear which probiotic combinations are most effective [6].

As we know, probiotic colonization that increases mucosal barrier function can alter the key components of intestinal inflammation and upregulate the immune system [7]. However, currently, the study about correlation of intestinal microbiome and inflammatory factors is still lacking, especially the coeffect of probiotic supplement on gut microbiome and inflammatory factors. We hypothesized that intestinal microbes may be involved in the pathogenesis of not only enteric disease like NEC but also systemic inflammatory processes.

In this study, we conducted a randomized, double-blind controlled trial in VLBW infants with a probiotic supplement in order to elucidate the effects of combined probiotics on gut microbial community and inflammatory factors.

## 2. Materials and Methods

### 2.1. Ethics Statement

All study procedures were reviewed and approved by the Ethics Committee of Shenzhen Bao'an Maternal and Child Health Hospital. Informed consent was signed by the parents of each infant following the protocol approved by the Institutional Review Board (registry number: LL2014006).

### 2.2. Study Design and Sample Collection

This is a randomized, double-blind controlled trial. Preterm infants with gestational age (GA) ≤ 34 weeks and birth weight (BW) < 1500 gm admitted to the Shenzhen Bao'an Maternal and Child Health Hospital from Sep. 1, 2014 to Dec. 31, 2015 and who survived in NICU were enrolled after obtaining the parent informed consents. The preterm infants with severe asphyxia (stage III), fetal chromosomal anomalies, cyanotic congenital heart disease, congenital intestinal atresia, gastroschisis, omphalocele, active upper gastric intestinal bleeding, lacking/refused of parental consent, or those fasted for >3 weeks during the study period after birth were excluded.

### 2.3. Randomization, Allocation Concealment, Blinding, and Follow-Up

Enrolled VLBW infants were randomized into two groups: the probiotic (PB) group, which was treated with combined probiotics, and the placebo (PL) group, which was treated with placebo. Randomization was performed using a sequentially numbered computerized randomization algorithm. The allocation to treatment was concealed by the principal investigator according to sequential numbers before starting. The drugs were supplied by Glac Biotech Co. Ltd. and were identical in the package, size, and shape. Also, the drugs were labeled B and C before shipment. The drugs were added to breast milk or formula before feeding by senior nurses who were not involved in the care of these infants. The nurses and doctors involved in managing infants did not know the drug content until the end of the study. All enrolled infants were cared for and followed up by the attending doctor up until 36 weeks post menstrual age or discharge.

### 2.4. Feeding Guideline and Intervention

All enrolled infants were considered for initial feeding within 24 hours after birth depending on the gestational age and birth weight. The mother's breast milk was preferred, follow by donor milk, then preterm formula. On the first day, minimal breast milk or formula was given every 2-4 hours depending on the feeding tolerance. The amount of feeding was increased slowly if tolerated, with increments of no more than 20 mL/kg per day per feeding. An oral intake of 100 mL/kg per day was defined as complete enteral feeding. Feeding was stopped if there was any sign of feeding intolerance, including the presence of gastric aspirate in an amount that was more than one half of the previous feeding, twice, or abdominal distension.

In the meanwhile, enrolled infants received treatment as part of either the probiotic group: breast milk or formula with combined probiotic (containing *L. plantarum* LK006 20%, *B. longum* LK014 40%, and *B. bifidum* LK012 40%; each probiotic capsule contains 500mg of 5^10^ colony-forming units (CFU), supplied by Glac Biotech Co. Ltd.), or the placebo group: receiving 1 mL of a 5% glucose solution. The total of 500 mg probiotics (contains 5^10^ CFU) per day or glucose was given by nasogastric tube within 4 h after birth. 250 mg per dose was added twice daily to the breast milk or formula until to 36 weeks post menstrual age.

The characteristics, clinical information, and lab data were extracted from our medical records.

Fecal specimens were collected in 30-50 g at 14 and 28 days of life and then transported immediately to the laboratory on ice and stored at -80°Cfor further studies.

The primary outcome was to evaluate the change of gut microbiota in VLBW infants after the combined probiotic supplement. The secondary outcome was to analyze the correlation gut microbial composition and levels of cytokines for elucidating the beneficial effects after probiotic supplement.

### 2.5. DNA Extraction

Genomic DNA was extracted from each fecal sample using the QIAamp Fast DNA Stool Mini Kit (QIAGEN, Germany) according to the manufacturer's instructions. The amount of DNA was determined by a NanoDrop 2000 UV-Vis spectrophotometer (Thermo Scientific, USA). Integrity and size of DNA were checked by 0.8% (*w*/*v*) agarose gel electrophoresis in 0.5 mg/mL ethidium bromide. All DNA samples were stored at −20°C prior to further processing.

### 2.6. 16S rRNA Gene Sequencing

The bacterial forward primer 5′-CCTACGGGRSGCAGCAG-3′ and reverse primer 5′-GGACTACVVGGGTATCTAATC-3′ were used to amplify the V3-V4 hypervariable regions of the 16S rRNA gene in each sample. The concentration of DNA libraries was quantified using PicoGreen DNA Assay (Invitrogen, USA). Pooled DNA library was diluted to 10 pM and denatured in 0.2 N NaOH and mixed with PhiX control library (Illumina Inc., USA). The DNA library was sequenced with an Illumina MiSeq sequencer (Illumina Inc., USA).

### 2.7. Bioinformatics and Statistical Analyses

The Quantitative Insights Into Microbial Ecology pipeline was employed to process the sequencing data (QIIME ver. 1.9.0, http://www.qiime.org/). Paired-end reads were merged using PANDAseq, sequences were denoised using USEARCH (ver. 8.0.1623), and chimera was checked with UCHIME26. Operational Taxonomic Units (OTUs) were picked using UCLUST at 97% similarity, and representative sequences were generated. Sequences were aligned with PyNAST using the Greengenes database and taxonomy assigned to the lowest possible taxonomic level using the Ribosomal Database Project Classifier at an 80% bootstrap value threshold. OTUs found in more than 50% of samples were retained. The numbers of sequences were normalized for further analyses.

In-group bacterial diversity (i.e., *α*-diversity) was assessed with ACE, Chao, Shannon, and Simpson indexes. Weighted Fast UniFrac principal coordinate analysis (PCoA) based on OTUs was performed to provide an overview of gut microbial dynamics in response to probiotic and placebo treatments. Between-group bacterial difference (i.e., *β*-diversity) was examined using a standard *t*-test. Pearson's correlation coefficient was calculated to evaluate the association between bacteria abundance and cytokine levels. Statistical analyses and data visualization were performed using R software package (version 3.4.2).

## 3. Results

### 3.1. Characteristics of Participants in the Study

**Figure 1:**
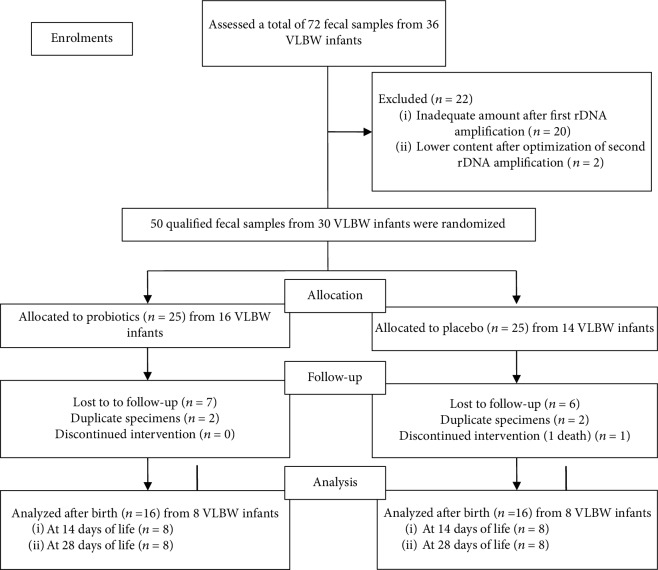
The flowchart of the present study.

During the study periods, a total of 72 fecal specimens collected from 36 VLBW infants on days 14 and 28 of life were collected. Of all these, 20 fecal samples were excluded completely due to inadequate amount after Meta rDNA amplification, and 2 specimens were further excluded because of lower content despite optimization of second Meta rDNA amplification. Finally, 50 fecal specimens extracted from 30 preterm VLBW infants were qualified and underwent 16S rRNA gene sequencing. During the follow-up periods, 18 fecal specimens were further excluded due to the lack of samples at 14 days or 28 days of life and duplication. Finally, 32 fecal specimens extracted from 16 preterm VLBW infants were analyzed. The flowchart of this study was shown in Figure 1.

All sixteen preterm VLBW infants were randomly divided into two groups receiving probiotic and placebo treatments (hereinafter termed PB and PL, respectively). The detailed demographic, clinical characteristics, feeding types, and antibiotic exposure of the two groups are summarized in Table 1. There are no differences between the two groups in terms of demographic data, clinical features, feeding types, and antibiotic exposure (*p* > 0.05).

### 3.2. Probiotic Treatment Affects Gut Microbiota in VLBW Infants

**Figure 2:**
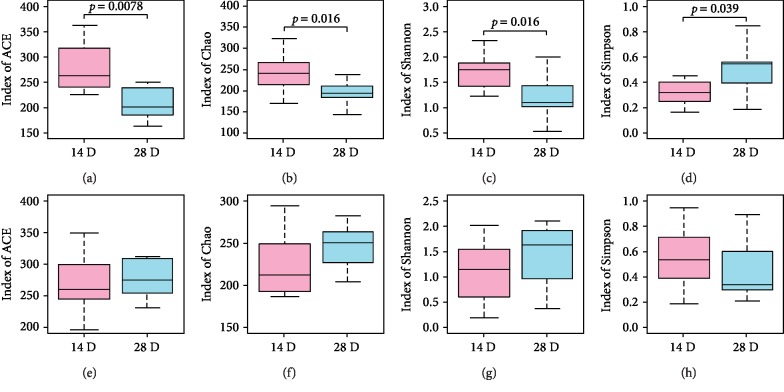
The changes of *α*-diversity in the PB group (a–d) and the PL group (e–h).

On the basis of high-quality reads that were obtained in sequencing, we identified a total of 597 Operational Taxonomic Units (OTUs) in all samples at 97% similarity level. ACE, Chao, Shannon, and Simpson indices were applied for analysis of *α*-diversity (Figure 2). In the PB group, the decreased ACE, Chao, and Shannon indices and the increased Simpson index on days 14 and 28 of life collectively demonstrated a reduced *α*-diversity after probiotic treatment. In contrast, no significant differences were detected on days 14 and 28 after placebo treatment in the PL group.

**Figure 3:**
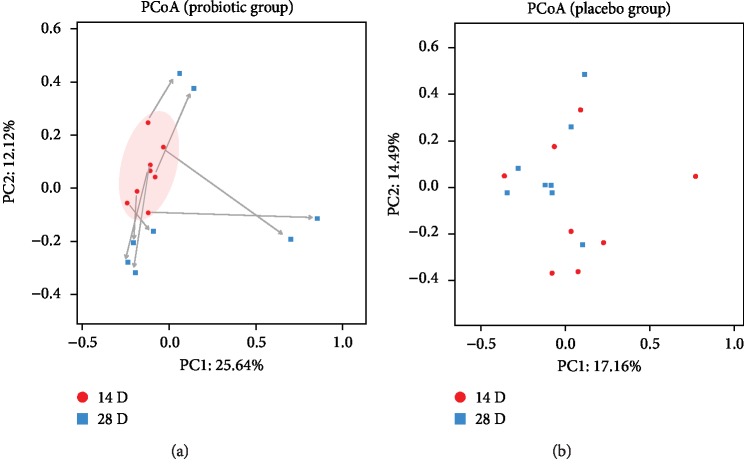
Principal coordinate analysis (PCoA) on the fecal microbiota of the PB group (a) and the PL group (b). 14-day and 28-day samples are shown in separate panels to emphasize the temporal difference.

Analysis of *β*-diversity was also performed by calculating the unweighted UniFrac distances between individual samples. Principal coordinate analysis (PCoA) plots demonstrated that the samples aggregated to form a cluster on day 14 of life, which dispersed on day 28 in the PB group (Figure 3(a)). However, no evident change was observed on days 14 and 28 of life in the PL group (Figure 3(b)).

### 3.3. Correlation between Gut Microbial Composition and Levels of Cytokines

**Figure 4:**
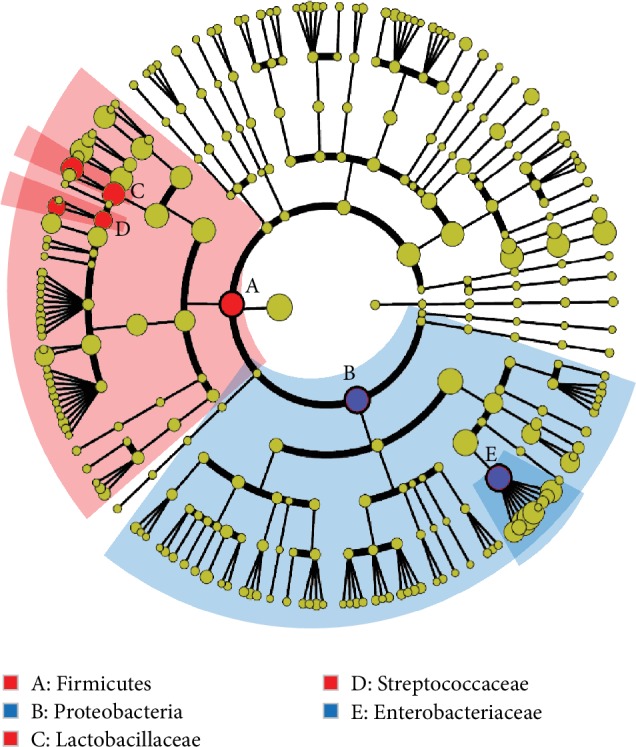
Cladogram generated by the LEfSe method indicating differences in the bacterial taxa between 14-day and 28-day samples from the PB group. Nodes in red indicate bacteria that were enriched on 28-day neonates, while nodes in blue indicate bacteria that were enriched on 14-day neonates.

At various taxonomic levels, gut microbiota composition was compared between 14- and 28-day fecal samples in the PB group. A cladogram representing the microbiota structure and the predominant bacteria was generated by linear discriminant analysis effect size (LEfSe) method (Figure 4). At the phylum level, *Firmicutes* was significantly more abundant in the gut microbiota on the 28-day samples than the 14-day ones. And the relative abundance of *Proteobacteria* was decreased on 28 days of life. At the family level, *Streptococcaceae* and *Lactobacillaceae* became prevalent on 28 days of life, while the relative abundance of *Enterobacteriaceae* was reduced in the meantime.

**Figure 5:**
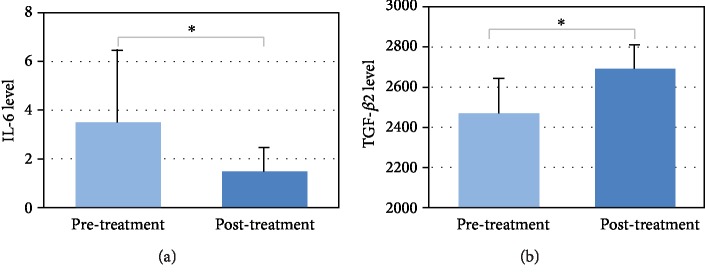
The significant changes in serum levels of IL-6 (a) and TGF-*β*2 (b) in the PB group.

To determine whether probiotic treatment relieved the symptoms of VLBW infants, we also evaluated the cytokine levels in the serum samples from the PB group. On day 28 of life, a decrease of IL-6 (paired Student's *t*-test *p* value = 0.026) and an increase of TGF-*β*2 were observed (paired Student's *t*-test *p* value = 0.011) in the PB group (Figure 5). On the other hand, no significant change in cytokine levels was detected in the PL group (data not shown).

**Figure 6:**
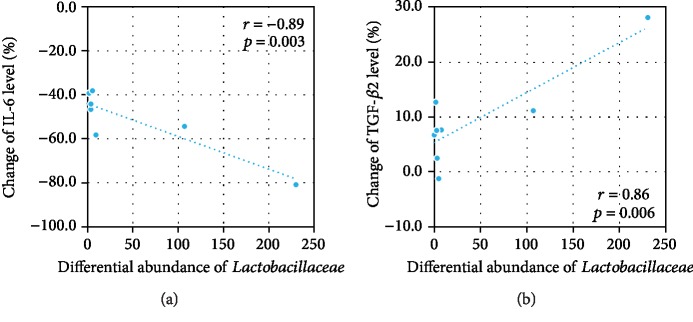
Correlation between the differential abundance of bacteria and the change of cytokines. Pearson's correlation coefficient (*r*) and two-tailed probability (*p*) were used to evaluate statistical importance.

We finally evaluated correlations among the relative abundances of various bacteria, IL-6, and TGF-*β*2. Among the differential bacteria between the 14- and 28-day samples, the differential abundance of *Lactobacillaceae* was significantly correlated with the changes of IL-6 (Figure 6(a)) and TGF-*β*2 (Figure 6(b)). An increase in the abundance of *Lactobacillaceae* was accompanied by a reduction of IL-6 and an elevation of TGF-*β*2.

## 4. Discussion

Cumulating data suggest that the human gut microbiota has profound influence on host metabolic disorders [8]. A comparison and investigation on the bacterial diversity of gut microbiota is essential for understanding the etiologies of preterm low birth weight infants and for developing potential treatment strategies. In this study, we explored the efficacy of combined probiotics on the bacterial diversity, the community structure, and the immune system in VLBW infants. To our knowledge, this is the first time to study the changes and correlation of gut microbiota and inflammatory factors simultaneously in VLBW infants with an oral combined probiotic supplement.

LEfSe identified a series of differentially abundant taxons between 14-day and 28-day samples from the PB group. Our results were mostly reasonable and in line with the experiment design or published findings. First, the combined probiotics tested in this study contain *L. plantarum*. As a result, the proportions of the *Lactobacillaceae* family unsurprisingly increased in the PB group [9]. Second, at the phylum level, the increase of *Firmicutes* and the decrease of *Proteobacteria* after probiotic treatment tend to be beneficial, since it has been reported that fecal microbiome from preterm infants with necrotizing enterocolitis had increased relative abundances of *Proteobacteria* and decreased relative abundances of *Firmicutes* [10, 11]. Such bacterial imbalance could be reversed by probiotics in the present study. Third, at the family level, the increase of *Streptococcaceae* and *Lactobacillaceae* and the decrease of *Enterobacteriaceae* can be readily explained by the previous findings that *Enterobacteriaceae* are predominant in preterm VLBW infants while *Streptococcaceae* and *Lactobacillaceae* are predominant in full-term infants [12]. For that reason, a decrease in *α*-diversity after probiotic treatment occurred due to the reduced relative abundance of harmful bacteria, whose share in the microbiome was taken over by probiotic species.

In the PB group, probiotic treatment was accompanied by a decrease of IL-6 and an increase of TGF-*β*_2_ in the serum. Consistent with previous findings [13], such changes of cytokines implied this protective effect of TGF-*β*, particularly the TGF-*β*_2_ isoform, via suppression of macrophage inflammatory responses in the developing intestine [14].In the microbiological studies of VLBW infants that have been performed thus far, little attention has been paid to the correlation between changes in the microbiota and cytokine levels. Thus, the detailed mechanism of probiotics on preterm VLBW infants remained largely unclear. In the present study, we found that *Lactobacillaceae* was significantly correlated with the serum levels of IL-6 and TGF-*β*_2_. This suggested that this bacterial family is closely related to the pathology of VLBW infants. In view of this, the abundance of *Lactobacillaceae* and other well-known probiotic genera (e.g., *Bifidobacteria*) may serve as a biomarker of the efficacy of probiotic treatment.

In spite of new discoveries, our study has certain limitations. First, the sample size was relatively small. However, our study is unique in having a serial assessment of probiotic treatment and gut microbiota. Furthermore, the changes in major bacterial phyla observed in this study were in accordance with the current understanding of the effect of those microbes. Second, all subjects were ethnic Chinese and the findings may not be directly extrapolated to other populations. Further confirmatory studies on other ethnic groups are welcomed to understand the interpopulation variation in gut microbiota.

In summary, we identified a number of bacteria with differential abundance upon probiotic treatment, which were not found in the placebo group. Based on these, future metagenomic studies involving larger preterm VLBW cohorts may improve probiotic therapies and elucidate the causal relationships between gut microbiota and preterm VLBW infants.

## Figures and Tables

**Table 1:** The demographic and clinical features of mothers and their infants between two groups.

	PB group (*n* = 8)	PL group (*n* = 8)	OR (95% CI)	*p* value
Maternal features				
Age (mean ± SD)	27.5 ± 3.8	28.6 ± 7.5		0.7
C-section (*n*, %)	5 (62.5)	3 (37.5)	2.8 (0.4~21.0)	0.6
PROM (*n*, %)	4 (50)	1 (12.5)	7.0 (0.6~86.3)	0.3
Pregnancy hypertension (*n*, %)	1 (12.5)	1 (12.5)	1.0(0.0~19.4)	1.0
Placenta previa (*n*, %)	2 (25)	0 (0)	0.4 (0.2~0.8)	0.5
Antibiotics before delivery (*n*, %)	1 (12.5)	0 (0)	0.5 (0.3~0.8)	1.0
Neonatal features				
Gestational age (weeks)	29.3 ± 1.3	30.4 ± 1.6		0.2
Birth weight (grams)	1176 ± 164	1326 ± 193		0.1
Male (*n*, %)	6 (75)	3 (37.5)	5.0 (10.6~42.8)	0.3
SGA (*n*, %)	1 (12.5)	1 (12.5)	1.0 (0.0~19.4)	1.0
1-minute Apgar score	8.5 ± 3.1	8.9 ± 1.6		0.8
5-minute Apgar score	9.4 ± 1.4	9.4 ± 0.9		1.0
Feeding types				
Exclusively formula	6 (75)	3 (37.5)		0.3
Breast milk plus formula	2 (25)	5 (62.5)		0.3
Antibiotic exposure (*n*, %)	7 (87.5)	5 (62.5)		0.6
Clinical complications				
CLD	4 (50)	3 (37.5)		1.0
IVH	2 (25)	0 (0)		0.5
ROP	1 (12.5)	0 (0)		1.0

CLD: chronic lung disease; IVH: intraventricular hemorrhage; ROP: retinopathy.

## Data Availability

The data used to support the findings of this study are currently under embargo while the research findings are commercialized. Requests for data (6/12months) after publication of this article will be considered by the corresponding author.

## Supplementary Materials

Supplementary 1 CONSORT 2010 checklist of information to include when reporting a randomized trial.

Supplementary 2 Figure S1: the changes of *α*-diversity (Shannon index) in the probiotic group and the placebo group.

Supplementary 3 Figure S2: a significant reduction in the percentage of neutrophils on day 28 in the PB group.

## References

[1] Stoll B. J., Hansen N. I., Bell E. F. (2015). Trends in care practices, morbidity, and mortality of extremely preterm neonates, 1993-2012. *JAMA*.

[2] Kong X., Xu F. D., Wu R. (2016). Neonatal mortality and morbidity among infants between 24 to 31 complete weeks: a multicenter survey in China from 2013 to 2014. *BMC Pediatrics*.

[3] AlFaleh K., Anabrees J. (2014). Probiotics for prevention of necrotizing enterocolitis in preterm infants.

[4] Athalye-Jape G., Rao S., Patole S. (2016). *Lactobacillus reuteri* DSM 17938 as a probiotic for preterm neonates: a strain-specific systematic review. *Journal of Parenteral and Enteral Nutrition*.

[5] Chang H. Y., Chen J. H., Chang J. H., Lin H. C., Lin C. Y., Peng C. C. (2017). Multiple strains probiotics appear to be the most effective probiotics in the prevention of necrotizing enterocolitis and mortality: an updated meta-analysis. *PLoS One*.

[6] Cassir N., Simeoni U., La Scola B. (2016). Gut microbiota and the pathogenesis of necrotizing enterocolitis in preterm neonates. *Future Microbiology*.

[7] Caplan M. S., Martin R. J., Fanaroff A. A., Walsh M. C. (2010). Neonatal necrotizing enterocolitis: clinical observations, pathophysiology, and prevention. *Fanaroff & Martin’s Neonatal-perinatal Medicine*.

[8] Warner B. B., Deych E., Zhou Y. (2016). Gut bacteria dysbiosis and necrotising enterocolitis in very low birthweight infants: a prospective case-control study. *The Lancet*.

[9] Mai V., Young C. M., Ukhanova M. (2011). Fecal microbiota in premature infants prior to necrotizing enterocolitis. *PLoS One*.

[10] Andrew J. (2016). Antibiotic perturbation of the preterm infant gut microbiome and resistome. *Gut Microbes*.

[11] Pammi M., Cope J., Tarr P. I. (2017). Intestinal dysbiosis in preterm infants preceding necrotizing enterocolitis: a systematic review and meta-analysis. *Microbiome*.

[12] Arboleya S., Ang L., Margolles A. (2012). Deep 16S rRNA metagenomics and quantitative PCR analyses of the premature infant fecal microbiota. *Anaerobe*.

[13] Maheshwari A., Schelonka R. L., Dimmitt R. A. (2014). Cytokines associated with necrotizing enterocolitis in extremely-low-birth-weight infants. *Pediatric Research*.

[14] Maheshwari A., Kelly D. R., Nicola T. (2011). TGF-*β*2 suppresses macrophage cytokine production and mucosal inflammatory responses in the developing intestine. *Gastroenterology*.

