# Supplementary material for: Beneficial Effects of Probiotic Treatment on Gut Microbiota in Very Low Birth Weight Infants
Source: Gastroenterol Res Pract. 2019 Oct 17;2019:3682836. doi: 10.1155/2019/3682836 (PMC6854177; doi:10.1155/2019/3682836)
Supplement: Supplementary 2 — Figure S1: the changes of α-diversity (Shannon index) in the probiotic group and the placebo group. [file 3682836.f2.docx]

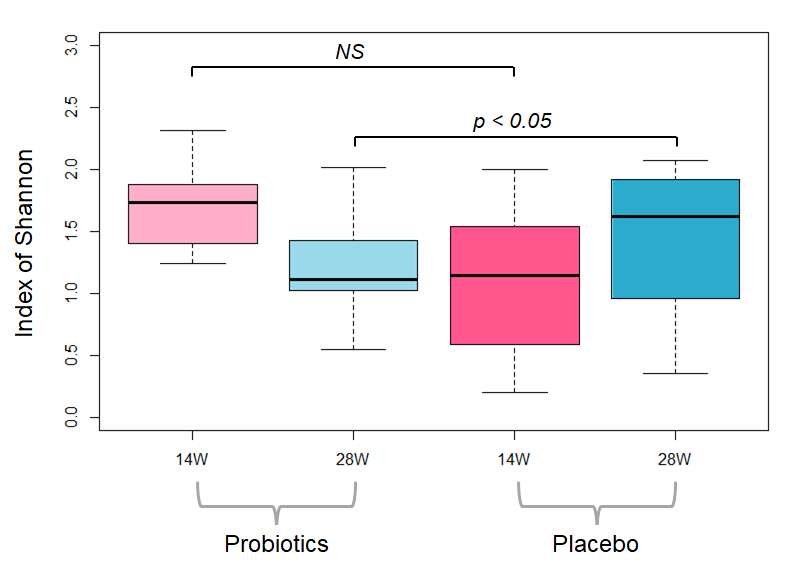


Figure S1. The changes of α-diversity (Shannon Index) in probiotics group and placebo group

In the Figure S1, we compared changes of α-diversity in day 14 and 28 of life in PB group with those in PL group. Compared with PL group, there is a significant decrease of α-diversity in day 28 (P<0.05) whereas no change of α-diversity in day 14 in PB group（shown in the Figure s1）.
