# Supplementary material for: Beneficial Effects of Probiotic Treatment on Gut Microbiota in Very Low Birth Weight Infants
Source: Gastroenterol Res Pract. 2019 Oct 17;2019:3682836. doi: 10.1155/2019/3682836 (PMC6854177; doi:10.1155/2019/3682836)
Supplement: Supplementary 3 — Figure S2: a significant reduction in the percentage of neutrophils on day 28 in the PB group. [file 3682836.f3.docx]

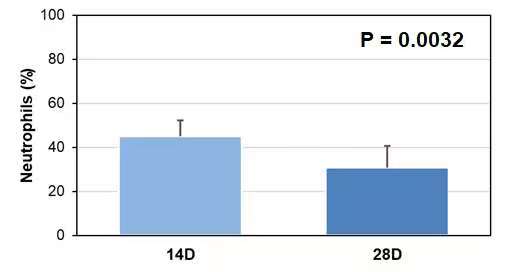


Figure S2 A significant reduction in the percentage of neutrophils on day 28 in PB group

Besides IL-6 and TGF-beta, we also evaluated correlations among the relative abundances of various bacteria and other variables, such as WBC count, percentage of neutrophils and CRP, etc. we found that the percentage of neutrophils was decreased from D14 to D28 during probiotics treatment (*P*<0.05) (shown in Figure S2). This indicated that the increase in the abundance of *Lactobacillaceae* was accompanied by a reduction of the percentage of neutrophils simultaneously. However, there were no different in the WBC count and CRP during probiotics treatment.
